# Taylor Swift versus Mozart: music preferences of C57BL/6J mice

**DOI:** 10.3389/fnbeh.2025.1668278

**Published:** 2025-10-15

**Authors:** Dominik Kamionek, Johann G. Maass, Claudia Pitzer, Christian P. Schaaf

**Affiliations:** ^1^Institute of Human Genetics, Heidelberg University Clinic, Heidelberg, Germany; ^2^Division of Genetics and Genomics, Boston Children’s Hospital, Boston, MA, United States; ^3^Interdisciplinary Neurobehavioral Core, Heidelberg University, Heidelberg, Germany

**Keywords:** music, C57BL/6J, mouse behavior, music preference, music intervention, environmental enrichment

## Abstract

**Introduction:**

Music has become an established complementary element of modern medicine, demonstrating beneficial effects towards various diseases such as dementia, hypertension, or chronic pain. Given its low cost and non-invasive nature, music-based interventions have been studied in both healthy mice and disease models over recent decades to examine potential effects in rodents. However, the selection of music in these interventions is based on prior reports and human preferences, without critically evaluating its relevance or perception in mice. Novel experimental approaches are needed to evaluate which type of music is preferred by mice.

**Methods:**

In this pilot study, we introduce a new experimental setup that can be used to analyze the music preferences regarding different genres and frequencies. Here, we present the first-ever evaluation of mouse music preferences by examining the behavioral responses of healthy C57BL/6J.

**Results:**

When given a choice between different musical conditions, mice spent comparatively less time in a chamber playing *Sonata for Two Pianos in D major, K.448* by Mozart, a piece regularly used in music-intervention studies of rodents. Further testing revealed that this behavioral response is independent of tone pitch.

**Discussion:**

These findings underscore the importance of species-specific tailoring of music selection towards therapeutic approaches. Our assay can be used to further broaden our understanding of murine music preferences and to analyze how mice respond to and perceive different auditory stimuli. Further studies are needed to systematically investigate murine music perception and preference across genres and exposure durations.

## Introduction

Music is considered an integral part of everyday life for most humans. Over the past decades, it has emerged as a therapeutic tool, widely used in medical interventions. It is valued for being cost-effective and associated with minimal side effects. Several meta-analyses have demonstrated beneficial effects of music therapy on various pathological conditions, including dementia (Lin et al., 2023), blood pressure (do Amaral et al., 2016), and chronic pain (Garza-Villarreal et al., 2017). However, music is a complex stimulus consisting of different core elements that define the character of a piece, such as melody, rhythm, harmony, or dynamics (Tzanetakis and Cook, 2002; Large et al., 2023; McFee et al., 2015). Therefore, music therapy in humans is often individualized according to the preferences of the patient, including the genre of music, duration of exposure, and sound pressure level (American Music Therapy Association, n.d.; de Witte et al., 2022). The perceived favorability of music was even found to be critical for its analgesic effect, irrespective of musical genre (Van der Valk Bouman et al., 2024). The multidimensional character of music poses a challenge for standardizing music interventions in both humans and animals. The same musical piece might produce significantly different effects across individuals, with an individual’s current affective state further influencing outcomes. While music interventions studies in humans can be tailored to the preference of the experimental subject, this is not possible in animal studies, making it essential to evaluate music preference and perception first.

In general, animal models are valuable tools in experimental research, as they enable detailed analysis of neurobiological mechanisms which cannot be directly studied in humans. To complement positive findings from human studies, the effects of music have been examined across various animal models to provide a multilevel perspective, integrating behavioral, physiological, and structural analyses. This approach allows researchers to explore how music influences brain structure, behavior, and homeostasis in both healthy animals and disease models, providing insights into translational applications (Kühlmann et al., 2018).

Numerous studies have shown a positive influence of music interventions on rodents, notably, enhanced neurogenesis and neuroplasticity (Lee et al., 2016; Fu et al., 2025; Xing et al., 2016b), improved learning ability (Xing et al., 2016a; Trzesniak et al., 2024), or a positive effect on blood pressure (Akiyama and Sutoo, 2011). Most of the studies examining music interventions in rodents used classical music as a stimulus (Kühlmann et al., 2018), especially the *Sonata for Two Pianos in D major, K.448* by Mozart is regularly used as an auditory stimulus in such experiments. Listening to this masterpiece was originally reported to enhance short-term, temporary spatial-reasoning in humans (Rauscher et al., 1993). The deduced so-called “Mozart effect” is nowadays considered to be more of a myth than an actual effect (Oberleiter and Pietschnig, 2023). Beyond that, rodents and especially mice have a hearing range that notably differs from humans (Heffner and Heffner, 2007). Rats can hear frequencies from around 500 Hz up to 64 kHz, while mice have a hearing range of about 2 kHz to 100 kHz. In comparison, the human auditory range spans from 20 Hz to 20 kHz (Heffner and Heffner, 2007). Most of *Sonata K.448* is below 1 kHz, only some tunes go up to 3 kHz. Thus, it is unlikely that mice can perceive the composition in the same manner as humans do. Adding to this problem, music preference is based on ethnological and individual factors.

While the importance of studying the effect of music on animals is indisputable, the type of music used in such studies needs to be carefully adjusted to the respective species.

Yet, the key parameters of music interventions are difficult to assess in rodents, given that the musical preference of mice is still not well understood within the scientific community.

In this pilot study, we aimed to develop a method capable of determining music preferences of wild-type C57BL/6J mice given a choice between four distinct genres: pop, electronic dance music (EDM), rock, and classical music.

Our study unexpectedly reveals that classical music, represented by *Sonata K.448* by Mozart, was associated with less time spent in the respective chamber when directly compared to the other presented compositions.

## Methods

### Animals

Mice at the age of 21 days were ordered from Jackson Laboratory and maintained at the Interdisciplinary Neurobehavioral Core of Heidelberg University, on a 12-h light/dark cycle. They had *ad libitum* access to food and water. Environmental conditions were controlled at 50–60% humidity and 22 °C (±2 °C). Mice were housed in Makrolon Type 2 cages with ABEDD LT-E-001 bedding and provided with environmental enrichment in the form of Crinklets Nest-Pads. Behavioral tests were performed during the light phase. Animal studies were approved by the Governmental Council Karlsruhe, Germany (G-174/21, principal investigator Claudia Pitzer).

We tested C57BL/6J mice, with a balanced distribution of male and female animals between postnatal day 28 and 37. Each animal completed the same experimental pipeline consisting of three experiments, as described in the following Music and Experimental Procedure section. C57BL/6J animals were chosen as this strain is frequently used in biomedical animal studies. The age of the mice was selected to lay the foundation for a subsequent music intervention study in mice with Bosch-Boonstra-Schaaf Optic Atrophy Syndrome (unpublished), also planned to start in juveniles. Additionally, this timing ensures that the auditory system is fully developed, as maturation is considered complete by this day (Sonntag et al., 2009).

### Mouse Disco

**Figure 1:**
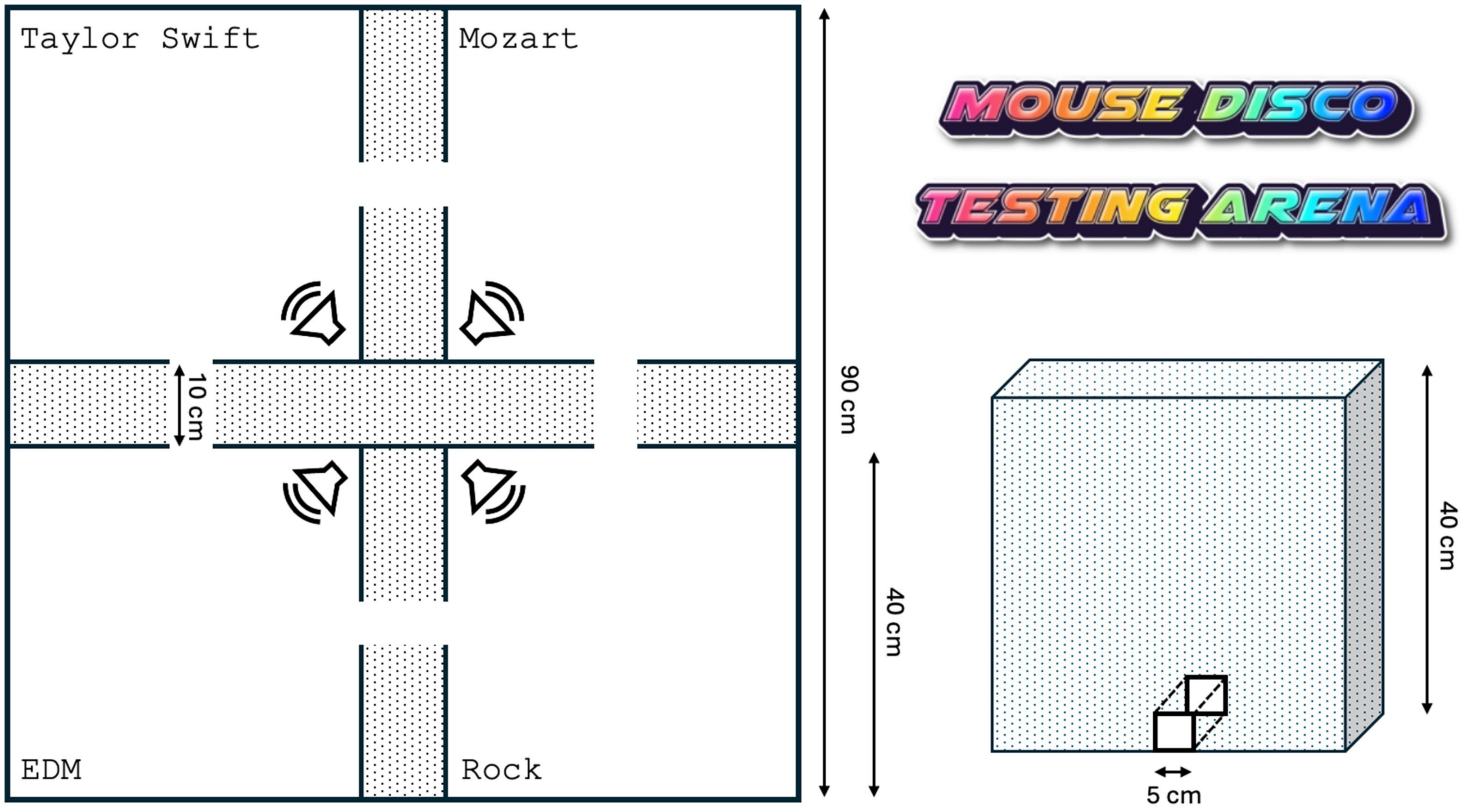
Layout of the Mouse Disco experimental setup. The arena consists of four rooms, in each room a different auditory stimulus (Taylor Swift, Mozart, Electronic dance music, and Rock, in alternation) is presented, delivered via appropriate speakers. The rooms are connected via tunnels. The walls are made of Open-cell Polyurethane foam (Schaumstoff-Center Vienerius, Bremen, Germany), while the ceiling of the disco area (not shown) is covered with acrylic glass to enhance sound isolation between rooms.

For the analysis of music and frequency preferences, we developed a new experimental setup. The testing arena (90 × 90 cm) consists of four rooms (each 40 × 40 cm) separated by Open-cell Polyurethane foam (10 cm thick, Figure 1). The foam was selected for its sound-absorbing properties, and sufficient sound isolation was confirmed (~10 dB cross-talk between chambers above an ambient background noise of 30 dB) using an UltraSoundGate condenser microphone (CM16/CMPA, Avisoft Bioacoustics) in combination with Avisoft-RECORDER software (Avisoft Bioacoustics). The four rooms were arranged in a square and interconnected by small tunnels (5 × 5 cm). The size of the opening was chosen to allow for the unhindered passage of the animals while maintaining the sound isolation between the rooms. The ceiling of the arena was made of thick (2 cm) acrylic glass to prevent the different melodies from travelling from one room to another while allowing our custom-developed mouse tracker to record the movement of the animals within the arena. The floor, the tunnels, and the walls were covered by white, disinfectable plastic.

Mice were habituated to the arena for 10 min before the music system was turned on. The movement of the mice was recorded for 60 min. A pre-test without music was done to evaluate whether mice showed a systematic preference for one of the rooms. Although this was not the case, we additionally alternated the music presented in each room between trials.

We are open to share and ship the setup should other research groups be interested.

### Music and experimental procedure

We tested four different musical genres, most of which have previously been used in the context of music therapy in rodents (Xing et al., 2016a; Xing et al., 2016b; Erken et al., 2008; Feduccia and Duvauchelle, 2008; Gao et al., 2016; Lu et al., 2010; Taheri et al., 2023; Meng et al., 2009). The first genre was classical music, represented by *Sonata for Two Pianos in D major, K. 448* (24 min), by W. A. Mozart, which was presented in a continuous one-hour loop. The second genre was classic rock, consisting of a curated mix of songs with a total duration of 22 min, also looped for 1 h. This mix included *Ain’t No Love in the Heart of the City* by Whitesnake, *Paint It Black* by Chris Parlowe, *Do not Fade on Me* by Firehouse, *Sharp Dressed Man* by ZZ Top, and *Bad, Bad Boy* by Nazareth. The third genre was electronic dance music (EDM), represented by the first 60 min of *The Very Best of Euphoric Dance: Breakdown 2001 – CD1* (Label: Telstar TV/BMG UK & Ireland). The final genre was contemporary pop music, consisting of a selection of Taylor Swift’s most-streamed songs on Spotify. This 18 min playlist, looped for 1 h, included *Cardigan*, *Shake It Off*, *Anti-Hero*, *Blank Space*, and *Cruel Summer*.

The amplitude of 65 dB was chosen based on protocols from previous music studies in rodents to ensure appropriate but not unpleasant sound pressure levels (Kühlmann et al., 2018). Testing was done on postnatal day 28.

Additionally, we tested both EDM and Mozart at different pitch levels: normal pitch, one octave higher, and two octaves higher on postnatal days 35 and 37, respectively. To achieve the pitch shifts, the music was transposed vertically using Audacity without altering the playback speed.

### Music system

The speakers were positioned 20 cm above the floor in the corner of the rooms facing the center, and projected their sound towards the opposite corner (Figure 1). This position proved to be ideal in terms of sound isolation. We used regular speakers for the tests of the four different musical genres. For the tests in which the frequencies were changed, we used an Ultrasonic Dynamic Speaker (Avisoft Bioacoustics, Berlin, Germany) and the Avisoft Portable Ultrasonic Power Amplifier. Prior to each session, the system was calibrated and checked to ensure an appropriate volume of 65 dB using the MK09 LCD digital sound level meter (Meterk, China).

### Statistics

Outlier analysis was performed using the ROUT method (Q value of 1%) to ensure robust exclusion of extreme data points for subsequent statistical analysis. All data points (including outliers) are shown in the figure. Animals that climbed onto the experimental set-up were excluded. Statistical comparisons were conducted using a one-way ANOVA, followed by *post hoc* T-tests for pairwise comparisons. To account for multiple testing, *p*-values of pairwise *post-hoc* T-tests were adjusted for multiple comparisons using the Benjamini–Hochberg procedure (*q*) (Benjamini and Hochberg, 1995). Statistical computations were performed using Python (libraries: pandas, numpy, and scipy.stats). Statistical significance was defined as *q* < 0.05 (adjusted *p*-values, Benjamini–Hochberg correction).

## Results

### The majority of *Sonata K.448* by Mozart is below the auditory range of mice

**Figure 2:**
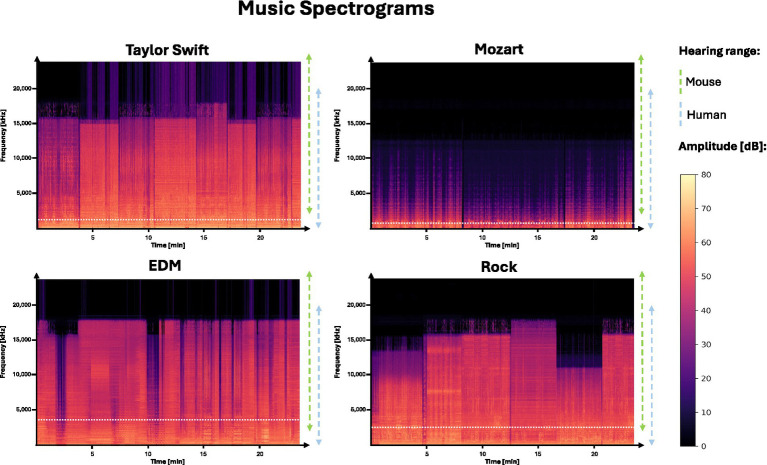
Spectrograms of the four different music tracks. Frequency-time-amplitude representations of four music types (Taylor Swift, Mozart, EDM, Rock) used in the experiment. Amplitude is shown in a color scale. Dashed lines indicate the hearing ranges of mice (green) and humans (blue). To allow an undistorted comparison of the music pieces, we selected or edited all music tracks to have a consistent length of 24 min, corresponding to the duration of *Sonata, K.448*. Horizontal white dashed lines indicate average frequency: Taylor Swift (1535.86 Hz), Mozart (820.20 Hz), EDM (3260.21 Hz), and Rock (2572.82 Hz).

Before assessing the music preferences of the mice, we conducted an objective analysis of the different musical stimuli. A first analysis of the spectrograms (frequency, amplitude, and time representation, calculated via the Fourier Transformation) shows significant differences in the frequency domains of the tracks (Figure 2). Mozart in particular presents a low frequency spectrum, mostly outside the hearing range of mice, which starts at 2 kHz. As such, this puts the usability of this particular track for music intervention studies in mice into question.

### Classical music and Taylor Swift songs are of higher complexity compared to rock and EDM songs

Additionally, we performed an in-depth quantitative analysis of the music complexity focusing on frequency changes (melody), rhythm and rhythm variation, harmonics, and dynamic patterns in the amplitude. This was done to ensure that the songs differ in objectively measurable structural features, beyond and independent of our culturally and individually biased human perceptions of music.

Classical music and songs by Taylor Swift exhibit higher musical complexity compared to Rock and EDM, as determined through objective computational analysis. Using the Librosa Python library (McFee et al., 2025), we analyzed the spectral, rhythmic, harmonic, and dynamic properties of selected tracks across these genres.

Before presenting our findings, it is important to note that the analysis is limited to the specific pieces discussed in this paper. Although we applied robust models, the true complexity of music is beyond mathematics. Thus, genre-specific conclusions should not be interpreted as value judgments, but rather as characterizations of selected measurable features.

We used the established Librosa Python library, focusing on their four dimensions of complexity, each corresponding to a key musical property (McFee et al., 2025). Audio features were extracted with Librosa and aggregated into the following indices:

1. Spectral complexity captures variation in timbre (i.e., melody) and is assessed through spectral contrast, representing differences between frequency bands.
2. Rhythmic complexity reflects tempo and beat variability, measured via fluctuations in the tempogram.
3. Harmonic complexity quantifies tonal diversity using chroma features, which indicate the relative intensity of the 12 pitch classes (e.g., C, C♯, D).
4. Dynamic complexity measures loudness variation, expressed as the standard deviation of root-mean-square energy over time.

Of each recording, 24 min were analyzed in one (Table 1). As the 24 min segment contains more than one song, and because combining multiple songs into a single analysis may inflate perceived complexity, we also computed complexity scores in 2 min segments (Supplementary Table S1). However, the genre ranking remained consistent.

**Table 1:** Spectral, rhythmic, harmonic, and dynamic complexity analysis.

Complexity indices				
Genre	Spectral	Rhythmic	Harmonic	Dynamic
Taylor	4.1989	0.2078	**0.2988**	**0.0977**
EDM	3.7641	0.2076	0.2908	0.0720
Mozart	**4.3344**	0.1728	0.2788	0.0333
Rock	3.7898	**0.2270**	0.2865	0.0529

Analysis of 4 complexity indices on the entire 24 min recordings of all music genres. Values are rounded to 4 decimal places. The highest complexity for each group is bold.

As expected, Mozart displays the highest spectral complexity, likely reflecting intricate melodic and timbral variation. Rhythmic complexity peaks in Rock, Taylor Swift and EDM. Because singing introduces additional layers of temporal variation, these genres show high rhythmic variation, as computed by the algorithm. While EDM initially appears rhythmically complex, its score decreases when analyzed in shorter intervals, indicating potential inflation from the simultaneous analysis of multiple songs (Supplementary Table S1).

Harmonic complexity did not show a consistent genre-wide trend, but is frequently highest in Taylor Swift tracks, indicating diverse pitch class usage. In contrast, dynamic complexity (variation in volume) is most pronounced in the songs by Taylor Swift and lowest in the music by Mozart.

**Figure 3:**
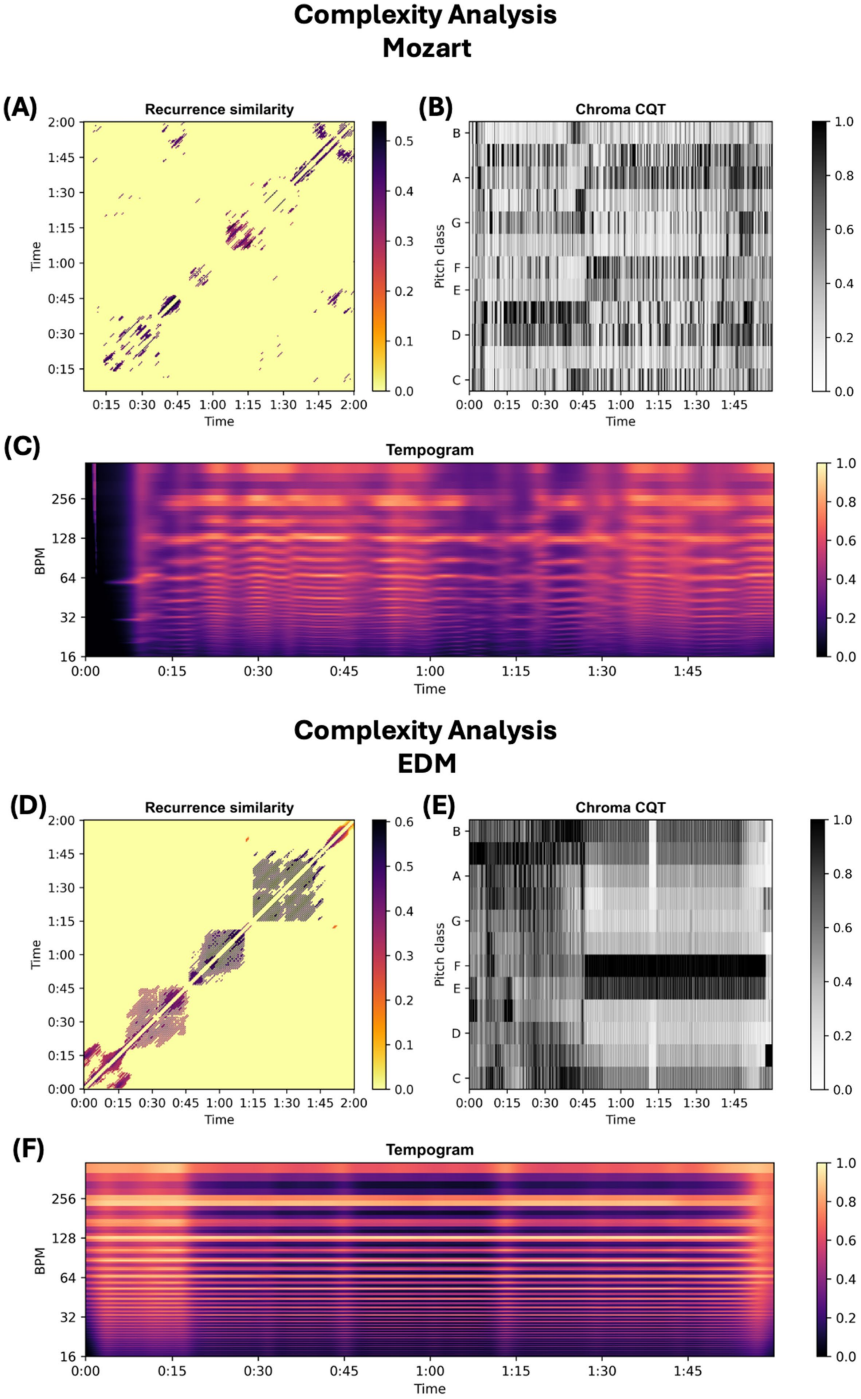
Comparative complexity analysis of Mozart and EDM using Librosa. Analysis was done using the Librosa Python library. Time is given in minutes. **(A,D)** Recurrence similarity (unitless): visualizes repeating patterns and structural self-similarity within the audio. **(B,E)** Chroma CQT (Constant-Q Transform): represents pitch class (C–B) intensity/normalized energy (unitless) over time. **(C,F)** Tempogram: depicts rhythmic periodicities in beats per minute (BPM) using autocorrelation (unitless).

To visualize these findings, we used Librosa to compute a recurrence similarity matrix, a Chroma Constant-Q Transform (CQT), and a tempogram on the first 2 min segment (visualizations of the entire 24 min intervals cannot be understood) (McFee et al., 2025). Figure 3 displays the first 2 min of Mozart and EDM pieces to highlight key structural differences; additional figures for Taylor Swift and Rock are provided in Supplementary material (Supplementary Figure S1). To keep this paper concise, the following text will focus on Mozart and EDM.

The recurrence matrix (Figures 3A,D) captures the temporal organization of repeated patterns. In EDM, large, block-like structures reflect high internal coherence and repetitive harmonic and timbral content. In contrast, Mozart lacks such blocks, suggesting minimal direct repetition and a greater diversity of motifs. The sparser and more irregular pattern in Mozart indicates a more complex and varied composition.

The Chroma CQT (Figures 3B,E) illustrates the distribution of pitch classes over time. While both Mozart and EDM exhibit considerable harmonic complexity, EDM shows a more patterned and repetitive structure, with sustained emphasis on a limited set of pitch classes. In Mozart, the chroma distribution is more dynamic and varied, reflecting a richer harmonic palette.

Finally, the tempogram (Figures 3C,F) shows rhythmic regularity by measuring the autocorrelation of onset events (e.g., drum hits, chords). A value of 1 denotes perfectly regular beat intervals. Although Mozart has lower rhythmic and dynamic complexity scores, the tempogram shows that EDM has stronger and more consistent rhythmic patterns, while Mozart displays greater rhythmic variation. This highlights that the rhythmic complexity score may be artificially elevated by vocal elements, which are absent in the first 2 min, and by the inclusion of multiple tracks in the EDM mix.

In conclusion, both the complexity score and the visual analysis indicate that *Sonata K.448* is structurally more complex than EDM across multiple musical dimensions.

### C57BL/6J mice spent less time in the Mozart compartment with no consistent differences between EDM, Taylor Swift, and Rock

To analyze the music preferences of the mice across the described compositions, we employed a two-step approach. In the first step, we conducted a direct comparison of the four musical conditions. Over 60 min, with a different type of music being presented in each compartment (at normal pitch), preference was assessed by measuring the time individual mice spent in each compartment.

**Figure 4:**
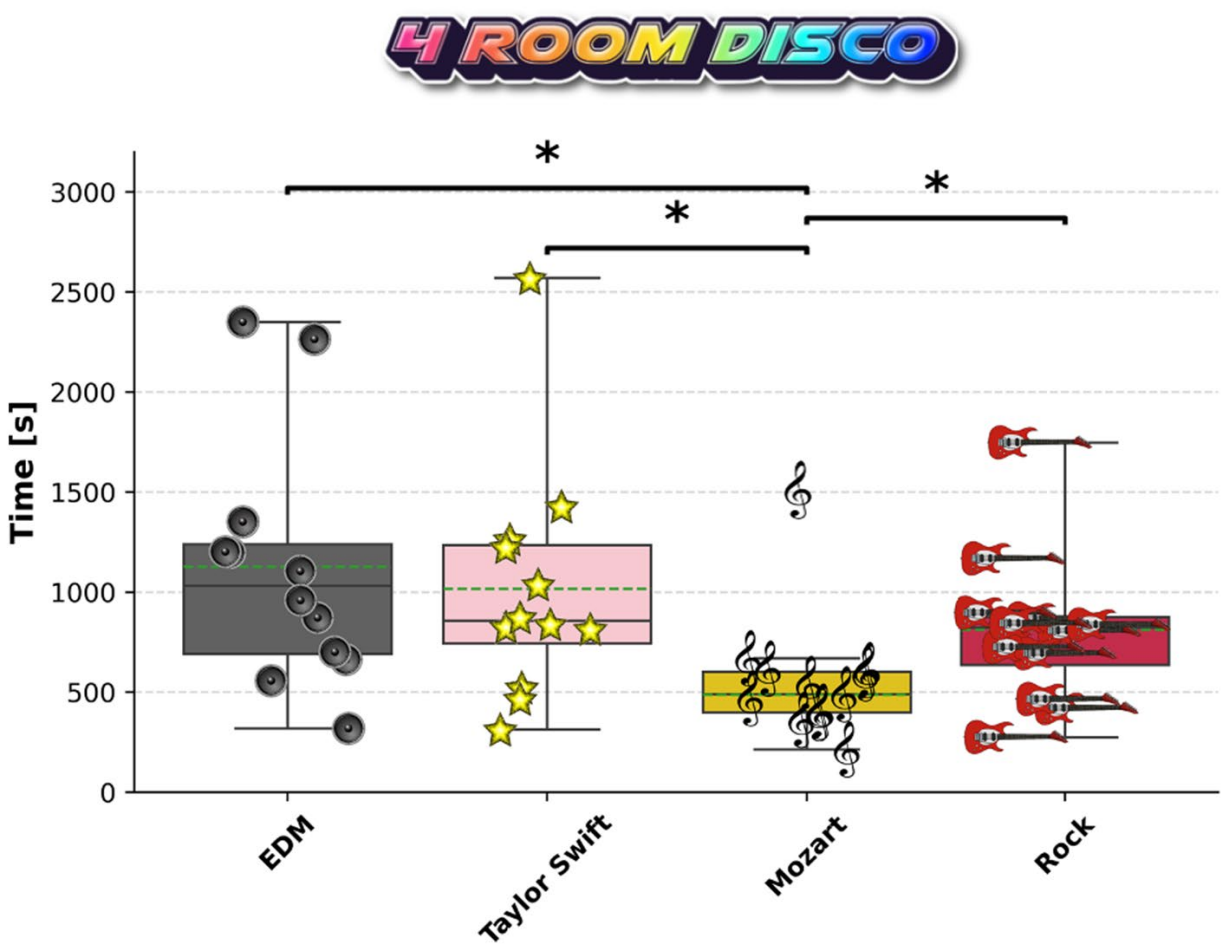
Test for chamber occupancy of C57BL/6J mice across musical conditions. Data points show the cumulative time spent in each room (EDM, Taylor Swift, Mozart, or Rock) over the 60 min test period. We tested 12 animals. Significances are analyzed using a one-way ANOVA followed by a *post hoc* T-test (homoskedastic) and Benjamini–Hochberg correction. *q < 0.05.

On average over the 60 min testing period, mice spent 1167.2 s in the EDM room, 1066.0 s in the Taylor Swift room, 487.7 s in the Mozart room, and 810.5 s in the Rock room. ANOVA revealed *p* = 0.0102 (F-statistic = 4.29), and *post hoc* T-tests (homoskedastic) showed a significant increase in the time spent in the EDM room compared to the Mozart room (raw *p* = 0.0026, *q* = 0.0152) (Figure 4). The same was observed for Taylor Swift vs. Mozart (raw *p* = 0.0051, *q* = 0.0152) and Rock vs. Mozart (raw *p* = 0.0197, *q* = 0.0393). Overall, the animals showed a reduced occupancy in the chamber playing Mozart, while no consistent differences were observed among EDM, Taylor Swift, and Rock.

### Mice do not show a difference in the time spent in compartments presenting different pitch conditions across EDM or Mozart

**Figure 5:**
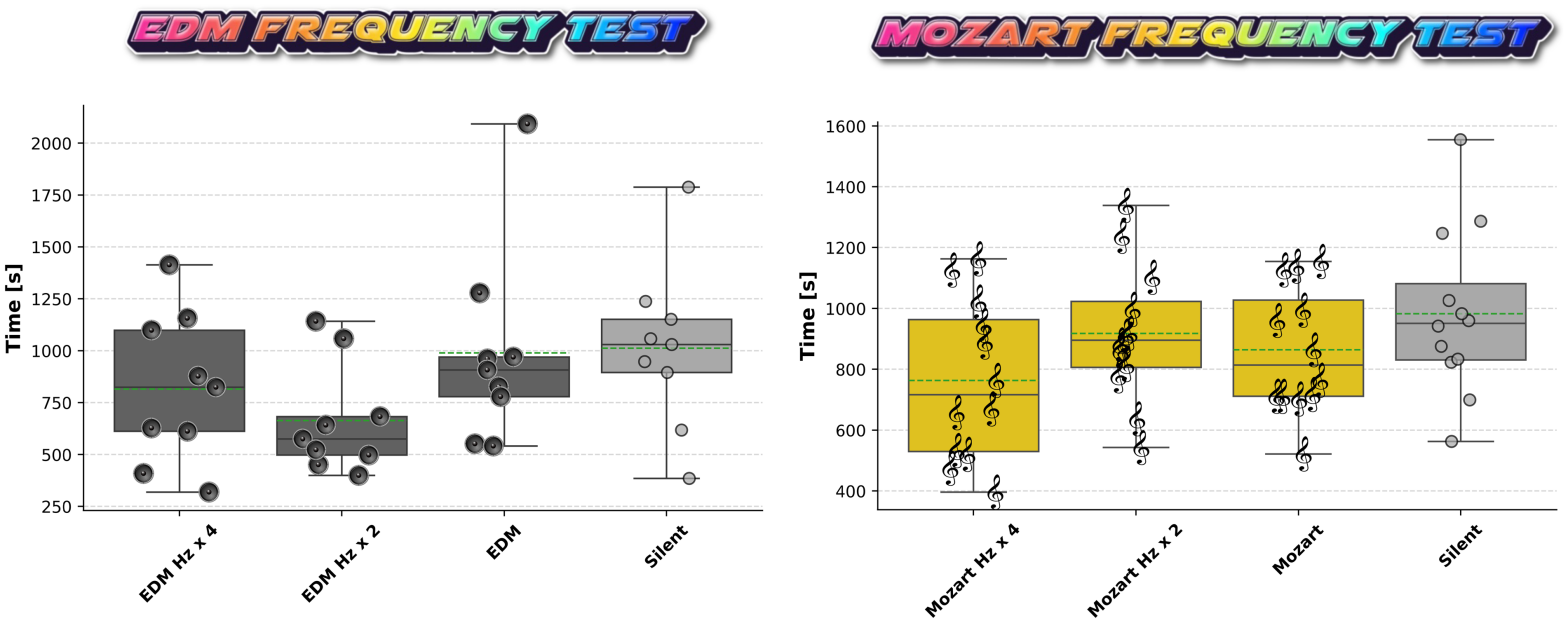
Test for chamber occupancy of C57BL/6J mice exposed to EDM/Mozart at different pitch conditions. Data points show the cumulative time spent in each room (EDM/Mozart normal, ×2, ×4 pitch, or silent) over the 60 min test period. We tested 9 animals for EDM and 12 for Mozart. One-way ANOVA showed no difference between the groups, *p* = 0.1934 (F-statistic = 1.67) and *p* = 0.1720 (F-statistic = 1.74), respectively.

In a second independent experiment, we specifically focused on the most and least preferred music types, identified based on average time spent in the rooms during the first experiment, evaluating them individually and with pitch adjustments. Since mice cannot perceive sounds below 2 kHz, we examined whether pitch alteration influenced their behavioral response. To allow the mice to experience a broader range of the musical content, the tracks were shifted up by one (Hz × 2) and two octaves (Hz × 4) and compared to a silent room and a room playing music at normal pitch. We focused on EDM and Mozart, based on the results from the initial test. One-way ANOVA revealed no significant differences between pitch conditions for either genre (EDM: *p* = 0.1934, F-statistic = 1.67; Mozart: *p* = 0.1720, F-statistic = 1.74). Additionally, there was no clear difference in time spent in the silent compartment. These results suggest that, despite theoretical expectations, increasing the pitch of the music does not alter the behavioral response in this paradigm (Figure 5).

## Discussion

To date, the scientific community lacks information regarding the music preference of mice. Despite this, music is recurrently used in murine therapeutic interventions, often without considering how mice actually perceive it. This highlights the need for novel approaches to understand how auditory stimuli affect mice.

Our findings in the music genre test indicate that wildtype C57BL/6J mice spent comparable amounts of time in chambers playing EDM, Rock and Taylor Swift, while showing reduced occupancy in the compartment presenting *Sonata K.448*. This behavioral response may suggest a relative avoidance of *Sonata K.448* when presented as an alternative, although definite statements on avoidance and preference require further testing, particularly using silence as a primary negative control. Our result deviates from initial expectations, as electronic dance music and rock music were primarily selected to serve as aversive stimuli. The assumption was based on a lack of therapeutic effect of rock music in previous studies (Cheng et al., 2024) and a generally agitated character of these music genres. The *Sonata K.448* by Mozart, in contrast, had been reported to show positive effects in numerous studies under behavioral, morphological, and physiological aspects (Kühlmann et al., 2018). A possible explanation for our result lies in the frequency properties of the music pieces. Given that mice have a hearing range in frequencies between 2 to 100 kHz, the frequency distribution of *Sonata K.448*, which is dominated by lower to mid-range frequencies by human standards, and an average around 820 Hz, may lie outside their optimal auditory sensitivity, potentially rendering it unappealing or even stressful, though speculative due to the absence of surveillance of physiological stress markers. The EDM mix, in contrast, has a wider spectrum of frequencies, with peaks exceeding 15 kHz, and an average of around 3,200 Hz, so mice are arguably able to perceive broader parts of the music than is possible in the Mozart composition.

Furthermore, as we have shown, the rhythm of EDM generally consists of repetitive and rhythmic beats with a clear periodicity. The music by Mozart, in contrast, is highly structured, complex, and harmonically rich. Previous work has demonstrated that, e.g., female mice are attracted to rhythmic and temporally regular acoustic courtship behavior (Perrodin et al., 2023). This may reflect an evolutionary adaptation to the environment in which complex sensory input is filtered to prioritize information relevant for survival. Accordingly, the rhythmic regularity and temporal clarity of EDM may be easier for mice to perceive, process, and interpret, potentially explaining the observed behavioral response, suggesting a relative preference of EDM over Mozart. Conversely, the relative avoidance of *Sonata K.448* over the other presented stimuli might stem not from aversion *per se*, but from the greater perceptual challenge posed by its complexity, suggesting that music with less temporal predictability may be less accessible or less behaviorally engaging for mice under these conditions.

Notably, the initial preference findings were not further supported in the frequency test. No significant differences in the time spent in either the music or the silent room were observed, which could indicate indifference rather than a true preference. Alternatively, this may also suggest that the potentially less attractive character of *Sonata K.448,* or the comparatively engaging character of EDM is not frequency-dependent. Furthermore, the animals did not spend more time in a music room than in the silent room, indicating that, under conditions of short-term exposure in a novel environment, music may not be more appealing than baseline silence. However, due to the brief duration of the experiment, it remains unclear whether this lack of preference reflects a general aversion to music or is simply a result of the specific experimental paradigm. Additional behavioral measures such as rearing, freezing, locomotor speed, transitions, grooming, and defecation could provide valuable context for interpreting these findings and help to distinguish true indifference from reduced occupancy to a particular music room. As the custom setup and tracking system only extracted positional data and did not store full-video recordings, these parameters could not be analyzed retrospectively. Further research should therefore implement these complementary measures.

Nevertheless, in a direct comparison under equal conditions, mice spent significantly less time in the chamber playing *Sonata K.448* compared to the other presented music stimuli, indicating a robust behavioral response. Music is perceived among humans variably, however, the variance in this result is remarkably low, highlighting the validity of the finding.

The use of a self-selection setup in our study, which allowed voluntary decision-making for a respective music genre, in combination with a habituation period prior to the measurement, likely minimized the stress-related bias.

It is important to point out that our results do not necessarily contradict previous findings on the positive influence of classical music on rodents. Numerous published studies are using rats in their experiments, which have a comparably lower frequency hearing range than mice. Additionally, recent research suggests that the analgesic effect of auditory stimulation in mice is rather based on a low signal-to-noise ratio of 5 dB than on harmonics (Zhou et al., 2022). It may be argued that auditory enrichment on a low scale can be sufficient to unfold its effect. Thus, it might not be necessary that the experimental animals hear the whole composition. Lastly, music preference should not be equated with therapeutic efficacy.

As this study is intended as a pilot study rather than a source of definitive conclusions, several limitations should be noted.

One limitation of our work is the short-term observation of animal behavior over an hour, whereas most of the therapeutic studies examine the effect of music intervention over a longer period. This constraint reflects the pilot nature of our study. Further research is necessary to examine how music preference develops over time and whether habituation or sensitization occurs with repeated exposure. Chronic and randomized exposure protocols would help to clarify true preference while controlling for novelty effects.

Another limitation is that, although minimized, cross-talk between rooms could not be completely eliminated; therefore, a potential influence of blended frequencies on our interpretations cannot be excluded.

To reduce the number of experimental animals, the same individuals participated in all three experiments. Resting periods of at least 48 h between sessions in the frequency experiment and a one-week interval between the music preference test and the subsequent frequency experiment were implemented to minimize adaptation and carry-over effects. In addition, chamber positions were randomized so that no mouse encountered the same musical conditions in the same compartment across sessions. Despite these precautions, we cannot fully exclude that animal reuse or order of experiments affected outcomes of the frequency experiments. Repeated exposure to the same auditory environment may have triggered familiarity or expectation, potentially biasing decision making by reducing novelty-driven engagement. Moreover, cumulative fatigue from multiple testing or exposure to potentially stressful musical conditions, could have contributed variability and should be considered when interpreting the results. However, the main findings from the preference test are unlikely to be influenced by these factors, as it was the first experiment conducted.

Finally, we based our study on a behavior paradigm. Further correlation with physiological markers, such as blood pressure, heart rate, or stress hormone levels, would provide a more comprehensive understanding of auditory preference in mice.

The presented assay provides a foundation for future research on auditory perception of music and its neurobiological influence in mice. Further experiments should include measurements of biological correlates of stress and well-being during music exposure and their correlation with behavior, as well as the evaluation of various ex vivo biological endpoints, with both acute and chronic exposure protocols considered. Moreover, explicitly linking behavior and neurobiological measures outcomes to specific acoustic features (e.g., rhythm, tempo tonal variability) would deepen our understanding of which musical components are most relevant for neurobiological effects. AI generated music with predefined rhythmic and spectral properties, potentially resembling the ultrasonic vocalizations of mice, could help to further explore the mechanisms underlying relative music preference. It is important to note that the music used in this paper is not fully representative of the genre labels, and that potential effects of music are likely driven by acoustic features rather than by genre itself; this should be considered in future research.

Overall, our study highlights the importance of critically selecting music used for therapeutic approaches in rodents. The common assumption that classical music is universally beneficial may not apply across all species and strains. Complex harmonics composed for human auditory perception are used in a substantial number of studies under the implicit premise that mice will perceive and derive benefit from them in a manner that is similar to humans. However, our findings indicate that, within our experimental setup, music by Mozart, specifically *Sonata K.448*, was comparatively less engaging than the other presented music stimuli, as measured by time spent in the compartments, suggesting that *Sonata K.448* may not be the most suitable for murine therapeutic approaches.

Hence, the auditory environment should always be tailored to the respective laboratory animal, as effects may be diminished by an inappropriate choice of music. This is also emphasized by Akiyama and Sutoo (2011) reporting that the lowering blood pressure effect of classical music by Mozart is dependent on the audibility of the music, as the effect vanishes when low-pass filters are applied to the music. Van der Valk Bouman et al. (2024) demonstrated that music from a preferred genre attenuates pain in humans, regardless of the specific genre, underscoring the importance of tailoring music to the individual preference of the experimental subject.

In conclusion, our pioneer study provides novel insights into the music preference of wildtype C57BL/6J mice, suggesting that *Sonata for Two Pianos in D major, K.448* by Mozart may be less engaging than selected compositions of EDM, rock or pop music. This finding challenges the assumption that music by Mozart is universally suitable for therapeutic interventions in mice, underscoring the importance of tailoring auditory stimuli to the respective experimental species. The unexpectedly higher occupancy in the EDM compartment relative to the Mozart compartment may be explained by the comparatively broader frequency spectrum and the simple, regular rhythmic pattern characteristics of EDM music. Additionally, we present a novel experimental assay for analyzing the music preferences in rodents, which can be used to evaluate different music types prior to future music intervention studies.

We acknowledge the limitations of our study and emphasize that a definitive conclusion regarding the music preferences of mice would require further analysis. Our work merely provides a platform for the scientific community to investigate this topic and suggests that Mozart may not be the preferred choice for mice.

This platform could also be valuable for exploring the therapeutic potential of music further which can serve as an effective and affordable tool without unwanted side effects, particularly as complementary support to established medical treatments. Numerous studies have reported positive effects of music in humans and rodents. However, we hypothesize that the beneficial effects may depend on music preference and, consequently, on music type.

Based on our findings, we advocate for a more nuanced and well-considered music choice in future therapeutic and behavioral studies involving laboratory animals, taking into account species-specific auditory preferences and sensitivities.

## Data Availability

The original contributions presented in the study are included in the article/Supplementary material, further inquiries can be directed to the corresponding author.
