# Supplementary material for: Taylor Swift versus Mozart: music preferences of C57BL/6J mice
Source: Front Behav Neurosci. 2025 Oct 15;19:1668278. doi: 10.3389/fnbeh.2025.1668278 (PMC12568665; doi:10.3389/fnbeh.2025.1668278)
Supplement: Supplementary file 1 [file Data_Sheet_1.PDF]

Supplementary Information

Taylor Swift versus Mozart: Music preferences of C57BL/6J mice.

Dominik Kamionek [1,†], Johann G. Maass [1,2,†,*], Claudia Pitzer [3], Christian P. Schaaf [1]

1 Institute of Human Genetics, Heidelberg University Clinic, Heidelberg, Germany
2 Division of Genetics and Genomics, Boston Children's Hospital, Boston, MA, USA
3 Interdisciplinary Neurobehavioral Core, Heidelberg University, Heidelberg, Germany

† Co-First
* Corresponding author: Johann G. Maass (email: johann.maass@childrens.harvard.edu)

Complexity Analysis
Rock

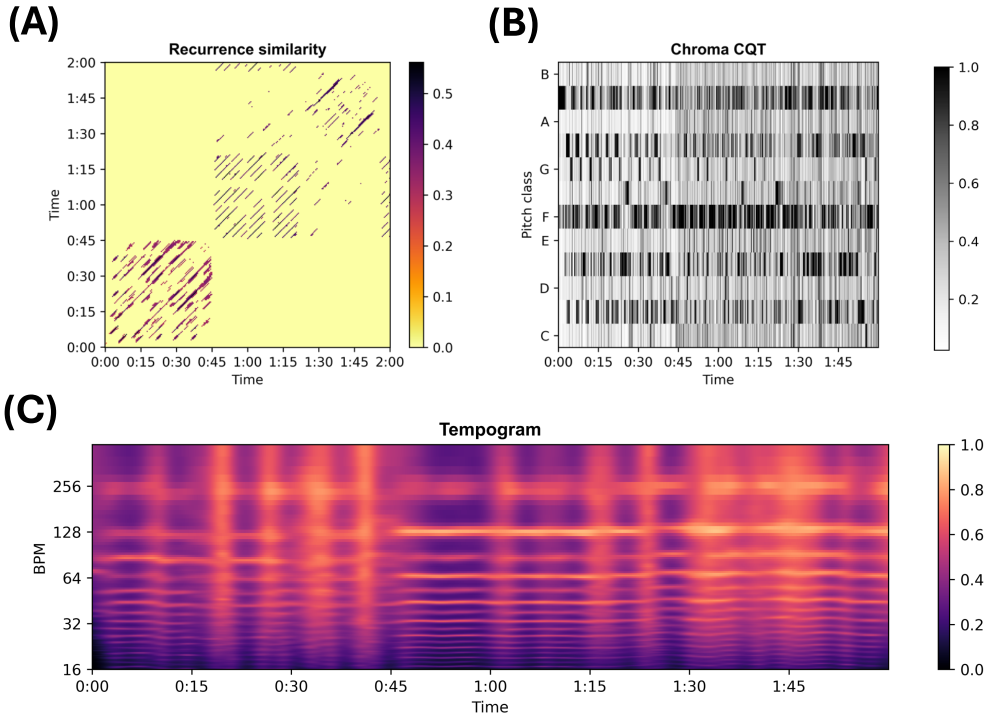

Complexity Analysis
Taylor Swift

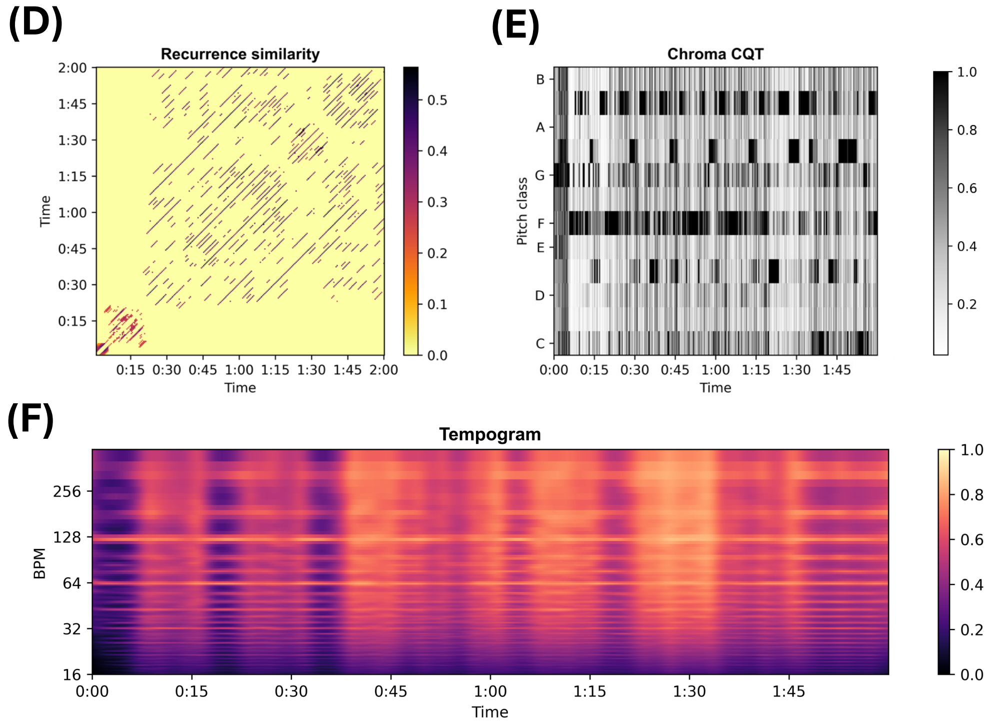

Figure S1: Comparative complexity analysis of Rock and Techno using Librosa.

Analysis was done using the Librosa Python library. Time is given in minutes. (A, D) Recurrence Similarity (unitless): Visualizes repeating patterns and structural self-similarity within the audio. (B, E) Chroma CQT: Represents pitch class (C–B) intensity/normalized energy (unitless) over time. (C, F) Tempogram: Depicts rhythmic periodicities in beats per minute (BPM) using autocorrelation (unitless).

Tables

Genre	Spectral	Rhythmic	Harmonic	Dynamic
Taylor	4.3644	0.2436	**0.2909**	**0.1004**
EDM	3.8177	0.2178	0.2570	0.0726
Mozart	**4.3489**	0.2222	0.2908	0.0285
Rock	3.7255	**0.2630**	0.2734	0.0467

Table S1. Spectral, rhythmic, harmonic, and dynamic complexity analysis.
Analysis of 4 complexity indices. The tracks were divided into segments of 2 min each, analyzed separately, and then averaged. Values are rounded to 4 decimal places. The highest complexity for each group is bold.

Full Overview of Statistical Analysis

Comparison	t-stat	p-value	BH-Corrected	Significant (BH)
**Music Genre Test**				
ANOVA Results:				
F-statistic: 4.29				
P-value: 0.0102				
Post Hoc Pairwise T-Tests:				
EDM vs Taylor Swift	0.38	0.7051	0.705125	False
EDM vs Mozart	3.44	0.0026	0.015171	True
EDM vs Rock	1.56	0.1333	0.199934	False
Taylor Swift vs Mozart	3.15	0.0051	0.015171	True
Taylor Swift vs Rock	1.18	0.2503	0.300327	False
Mozart vs Rock	-2.54	0.0197	0.039322	True
**EDM Frequency Test**				
ANOVA Results:				
F-statistic: 1.67				
P-value: 0.1934				
Post Hoc Pairwise T-Tests:				
EDM Hz x 4 vs EDM Hz x 2	1.02	0.3240	0.469502	False
EDM Hz x 4 vs EDM	-0.88	0.3913	0.469502	False
EDM Hz x 4 vs Silent	-1.11	0.2853	0.469502	False
EDM Hz x 2 vs EDM	-1.81	0.0885	0.265501	False
EDM Hz x 2 vs Silent	-2.21	0.0422	0.253049	False
EDM vs Silent	-0.11	0.9130	0.913040	False
**Mozart Frequency Test**				
ANOVA Results:				
F-statistic: 1.74				
P-value: 0.1720				
Post Hoc Pairwise T-Tests:				
Mozart Hz x 4 vs Mozart Hz x 2	-1.54	0.1369	0.410622	False
Mozart Hz x 4 vs Mozart	-1.04	0.3089	0.463408	False
Mozart Hz x 4 vs Silent	-2.02	0.0562	0.337069	False
Mozart Hz x 2 vs Mozart	0.61	0.5486	0.548601	False
Mozart Hz x 2 vs Silent	-0.63	0.5343	0.548601	False
Mozart vs Silent	-1.21	0.2409	0.463408	False

Table S2. Overview of the statistical analysis

Summary of all statistical tests conducted for the three independent experiments in this paper. Analysis was performed using Python (libraries: pandas, numpy, and scipy.stats). T-test results were subsequently adjusted for multiple testing using Benjamini-Hochberg correction (BH).
